# The Impact of Comorbidities on Functional Outcomes After Rehabilitation in Stroke Patients

**DOI:** 10.3390/healthcare14070851

**Published:** 2026-03-27

**Authors:** Tijana Dimkić Tomić, Olivera Djordjević, Sindi Mitrović, Suzana Dedijer Dujović, Stefan Rosić, Ljubica Konstantinović, Aleksandra Vidaković

**Affiliations:** 1 Faculty of Medicine, University of Belgrade, 11000 Belgrade, Serbia; odordev@gmail.com (O.D.); sindizmitrovic@gmail.com (S.M.); suzanadedijer@yahoo.com (S.D.D.); ljkonstantinovic@yahoo.com (L.K.); 2 Clinic for Rehabilitation “Dr. Miroslav Zotovic”, 11000 Belgrade, Serbia; stefan.rosic.86@gmail.com

**Keywords:** stroke rehabilitation, comorbidity, functional recovery

## Abstract

**Background:** Comorbidities are common among stroke survivors and may substantially influence functional recovery during rehabilitation; therefore, in this study, we aimed to evaluate the impact of individual comorbidities on functional outcomes in stroke patients after inpatient rehabilitation. **Methods:** In this retrospective cohort study, we included 289 patients with first-ever ischemic or hemorrhagic stroke who had undergone inpatient rehabilitation and assessed functional outcomes using the Barthel Index (BI), gait speed, Berg Balance Scale (BBS), and Action Research Arm Test (ARAT) at admission, after three weeks, and at discharge. The impact of selected comorbidities, including hypertension, diabetes mellitus, depression, cardiomyopathy, peripheral arterial disease, hyperlipidemia, and atrial fibrillation, was analyzed using multivariable logistic regression. **Results:** Significant improvements were observed across all functional measures (*p* < 0.0001). Diabetes mellitus and depression were independently associated with poorer improvement in BI, while reduced improvement in gait speed was associated with higher National Institutes of Health Stroke Scale (NIHSS) score, older age, female sex, cardiomyopathy, atrial fibrillation, and depression. Cardiomyopathy was also associated with reduced balance improvement measured by BBS, while vascular comorbidities were linked to less favorable upper limb recovery. **Conclusions:** Inpatient rehabilitation leads to significant functional recovery after stroke; however, specific comorbidities adversely affect rehabilitation outcomes. Targeted assessment and management of metabolic, cardiovascular, and psychological comorbidities may enhance functional recovery in stroke patients.

## 1. Introduction

Stroke is a leading cause of adult disability worldwide, with more than one million people in Europe experiencing a stroke each year, resulting in a substantial medical, social, and economic burden [1,2]. Numerous factors influence prognosis and recovery afterwards, including demographic characteristics (age, sex, socioeconomic status), stroke-related factors (ischemic or hemorrhagic type, lesion location and size), type of acute care (general wards or specialized stroke units), severity of neurological deficits (upper and lower limb impairment, balance and gait dysfunction), and comorbidities [3,4,5]. Identifying key variables relevant to recovery and rehabilitation outcomes enables better planning and continuity of rehabilitation following the acute phase.

For patients with impaired ambulation and upper limb motor function, inpatient rehabilitation plays a crucial role, as stroke survivors frequently present with multiple chronic diseases that may influence recovery. Comorbidity is defined as a stable chronic disease present at the time of admission to a healthcare facility [6], and a greater number of comorbidities are generally associated with poorer functional outcomes following rehabilitation [7]. Previous studies reported varying prevalences of comorbidities among stroke patients, with the proportion of patients without comorbid conditions ranging from 6% to 40% [8,9]. The impact of individual medical conditions on functional recovery varies, and poorly controlled comorbidities may limit active participation in rehabilitation programs, negatively affecting outcomes. Optimal management of comorbidities may accelerate recovery, reduce healthcare costs, and shorten hospitalization.

Assessing the impacts of comorbidities on physical disability, rehabilitation duration, and quality of life is essential for stroke research and clinical practice. Several comorbidity indices, including Charlson, Liu, and Schwartz, combine cardiovascular, metabolic, cognitive, musculoskeletal, and other conditions [10,11,12]. While many studies focus on the number of comorbidities present, data on the impact of individual comorbidities on functional outcomes remain limited [13]. Moreover, findings on the relationship between comorbidities and functional recovery are inconsistent [9,12,14,15,16]. Identifying the key variables influencing functional recovery after stroke contributes to improved planning and efficient use of healthcare resources.

The aim of this study was to evaluate the impact of individual comorbid diseases on functional outcomes in stroke patients after inpatient rehabilitation.

## 2. Materials and Methods

### 2.1. Study Design and Setting

Our retrospective cohort study included 289 stroke patients who underwent inpatient rehabilitation at the Clinic for Rehabilitation “Dr Miroslav Zotović,” Belgrade, between January 2017 and December 2019. Inclusion criteria were first-ever ischemic or hemorrhagic stroke confirmed by computed tomography (CT) or magnetic resonance imaging (MRI) and age ≥ 18 years. Patients participated in a structured inpatient rehabilitation program consisting of one hour of physical and one hour of occupational therapy per day, five days per week. Speech therapy (1 h daily) was also provided when indicated. Kinesiotherapy included task-specific training, gait training, muscle strengthening, neuroplasticity-oriented exercises, and training for activities of daily living (ADL). The rehabilitation program was individualized according to the patient’s functional status and tolerance, with gradual progression in exercise intensity and task complexity during the rehabilitation period. Therapy protocols were supervised by experienced physiotherapists and occupational therapists to ensure treatment consistency.

Exclusion criteria were severe cognitive impairment preventing active participation in rehabilitation (e.g., dementia), pre-existing neurological disorders with motor impairment, and inability to tolerate physical exercise during rehabilitation.

This study was approved by the institutional ethics committee of the Clinic for Rehabilitation “Dr Miroslav Zotović,” Belgrade (approval code: 3-203/1).

### 2.2. Data Collection

Demographic and clinical variables collected included sex; age; stroke type (ischemic or hemorrhagic); severity at admission, as assessed using the National Institutes of Health Stroke Scale (NIHSS); localization; and side of hemiparesis/hemiplegia. NIHSS scores were categorized as follows: 0 (no stroke symptoms), 1–4 (minor stroke), 5–15 (moderate stroke), 16–20 (severe stroke), and 21–42 (very severe stroke) [17].

Medical data were obtained from patients’ medical records and the following comorbidities were analyzed: arterial hypertension (HTN), cardiomyopathy (CMP), diabetes mellitus (DM), atrial fibrillation (AF), hyperlipidemia (HLD), peripheral arterial disease (PAD), and depression. All were assessed upon admission and recorded as “presence/absence”. Comorbidity selection was based on previous research in stroke rehabilitation, their prevalence in the clinical population, and their potential impact on outcomes [12,13,14].

Functional outcomes were assessed using the Barthel Index (BI), Action Research Arm Test (ARAT), Berg Balance Scale (BBS), and gait speed measured over a 10 m walk. The cut-off values used for categorization of functional outcome measures were based on previously published and widely accepted clinical thresholds reported in the literature [18,19,20,21,22,23]. These thresholds correspond to clinically meaningful levels of functional independence, balance, upper limb function, and gait ability, and are commonly applied in stroke rehabilitation research. For the purposes of regression analyses, outcome variables were additionally dichotomized to facilitate clinically relevant interpretation and comparability with prior studies. The BI assessed independence in activities of daily living (0–100), with higher scores indicating greater independence, categorized into total (0–20), severe (21–40), moderate (41–60), mild (61–80), and minimal dependency (81–100) [18,19]. Gait speed was measured using the ten-meter walk test (m/s) and classified as household (<0.4 m/s), limited community (0.4–0.8 m/s), or full community ambulation (>0.8 m/s) [20]. Balance was assessed using the BBS and categorized into three clinically meaningful groups reflecting fall risk: high (0–20), moderate (21–40), and low fall risk (≥41) [21,22]. Upper limb function was evaluated using the ARAT and categorized into severe (0–20), moderate (21–40), and mild/no impairment (≥41) [23].

Repeated measurements of functional outcomes were conducted at three time points: t1 = baseline (upon admission), t2 = after three weeks of therapy, t3 = at discharge. These repeated measures allowed for the evaluation of changes in activities of daily living, gait speed, balance, and upper limb function over time.

### 2.3. Data Analysis

Descriptive and analytical statistics were used to analyze the data.

Between-group differences with categorical data were explored using the χ^2^ or Fisher’s exact test. To analyze categorical data from repeated measures, Cochran’s Q test was used.

One-way repeated-measures ANOVA was used to determine whether the means of functional outcome scores were significantly different between the three time points. Multiple pairwise comparisons were made between the time points with the Bonferroni multiple testing correction method, and then a two-way repeated-measures ANOVA was performed to determine whether a significant interaction existed between comorbidity variables and time on functional outcome scores.

To test the associations between comorbidity variables and functional outcomes, multivariable binary logistic regression was used, with the models constructed using a three-stage approach: First, predictors were selected based on clinical relevance and a univariate screening (*p* < 0.20). Second, we used a mixed (bidirectional) stepwise selection procedure, wherein the algorithm iteratively added and removed variables to minimize the Akaike Information Criterion (AIC). Third, the final model was evaluated for multicollinearity using the Variance Inflation Factor (VIF), with values > 5.0 indicative of significant collinearity. Model calibration was assessed via the Hosmer–Lemeshow goodness-of-fit test.

To control the inflation of the Type I error rate due to multiple comparisons, we applied Bonferroni multiple testing within the repeated-measures ANOVA, while within the multivariable logistic regression, model complexity was strictly controlled via AIC-based variable selection to minimize the risk of over-fitting and false-positive associations.

The sample size for the multivariable logistic regression was analyzed using the Events Per Variable (EPV) criterion. For the functional outcome gait speed, there were 132 events (improvement in subjects between t1–t3 time points) and 5 predictors, where 2 had 4 categories while the others had 2 categories, so the degrees of freedom was 9. According to calculations, the EPV was 14.67, which is higher than the traditional ‘rule of 10’.

Statistical data processing was performed using R Studio software (Version 1.4.1106, 2009–2021 Rstudio, PBC, Boston, MA, USA) with the significance threshold taken at 5%.

## 3. Results

### 3.1. General Characteristics of Participants

A total of 289 patients were included in this study (58% male; 42% female), with most (64%) aged between 55 and 75 years. Right-sided brain lesions were present in 54% of patients, left-sided in 42%, and bilateral in 4%. Subcortical stroke localization was the most common (45%), followed by cortical (24%), mixed cortical–subcortical (18%), cerebellar (8%), and lacunar strokes (5%). Ischemic stroke accounted for 88% of cases. Moderate stroke severity was observed in 66.8% of patients, while 30.2% had a minor stroke.

Hypertension was the most prevalent comorbidity (98%), followed by diabetes mellitus (82%) and hyperlipidemia (71.3%). Depression was identified in 64.5% of the patients, whereas atrial fibrillation, cardiomyopathy, and peripheral arterial disease were present in 16%, 19.4%, and 20%, respectively (Table 1).

### 3.2. Functional Outcomes over Time

Significant improvements were observed across all functional measures (BI, gait speed, BBS, ARAT) at baseline, three weeks, and discharge (Table 2). The results of one-way repeated-measures ANOVA demonstrated statistically significant differences in BI (F (367.6, 1.3) = 290.5, *p* < 0.0001), gait speed (F (333.5, 1.3) = 245.7, *p* < 0.0001), BBS (F (339.5, 1.3) = 300.3, *p* < 0.0001), and ARAT (F (339.5, 1.3) = 300.3, *p* < 0.0001). Post hoc analyses with Bonferroni’s correction confirmed significant pairwise differences (*p* < 0.0001).

### 3.3. Barthel Index and Comorbidities

Distribution of BI categories differed significantly over time (Cochran’s Q test, *p* = 0.0004), indicating functional improvement (Table 3). All candidate predictors had a VIF < 2.0, which indicated that multicollinearity was not significant (NIHSS = 1.02, age = 1.32, sex = 1.05, diabetes = 1.21, cardiomyopathy = 1.19, peripheral artery disease = 1.47, hyperlipidemia = 1.12, hypertension = 1.02, atrial fibrillation = 1.29, and depression = 1.03). Through multivariable logistic regression, we identified sex, diabetes mellitus, and depression as independent predictors of BI improvement (Table 4): female sex was associated with lower odds of improvement (OR = 0.70; 95% CI: 0.43–0.94; *p* = 0.046), while absence of diabetes mellitus (OR = 1.73; 95% CI: 1.09–3.46; *p* = 0.047) and absence of depression (OR = 2.43; 95% CI: 1.98–3.68; *p* = 0.0005) were positive predictors of functional recovery (Table 4).

### 3.4. Gait Speed and Comorbidities

Patients were categorized into three groups based on their ten-meter gait speed, with distribution across categories differing significantly over time (Cochran’s Q test, *p* < 0.0001), reflecting functional improvement (Table 5).

All candidate predictors had a VIF < 2.0, which indicated that multicollinearity was not significant (NIHSS = 1.04, age = 1.36, sex = 1.05, diabetes = 1.27, cardiomyopathy = 1.15, peripheral artery disease = 1.42, hyperlipidemia = 1.11, hypertension = 1.16, atrial fibrillation = 1.25, and depression = 1.06).

Through multivariable logistic regression, we identified NIHSS score, age, sex, cardiomyopathy (CMP), and depression as significant predictors of gait speed improvement (Table 6): higher NIHSS scores, older age, and female sex were associated with lower odds of gait speed improvement, while absence of cardiomyopathy and absence of depression were associated with higher odds of gait speed improvement.

### 3.5. Berg Balance Scale and Comorbidities

BBS scores improved significantly across all time points (Cochran’s Q test, *p* < 0.0001), and so patients were divided into three score categories based on BBS scores, with the distribution differing significantly over time, indicating progressive balance improvement (Table 7).

All candidate predictors had a VIF < 2.0, which indicated that multicollinearity was not significant (NIHSS = 1.01, age = 1.38, sex = 1.06, diabetes = 1.25, cardiomyopathy = 1.18, peripheral artery disease = 1.46, hyperlipidemia = 1.09, hypertension = 1.14, atrial fibrillation = 1.29, and depression = 1.04).

Through multivariable logistic regression, we identified cardiomyopathy (CMP) as the only significant predictor of BBS improvement; absence of CMP was associated with higher odds of balance improvement (OR = 1.95; 95% CI: 1.04–3.76).

### 3.6. Action Research Arm Test (ARAT) and Comorbidities

ARAT scores were categorized into three levels of upper limb function, with the score distribution differing significantly across pre-, re-, and post-test assessments (Cochran’s Q test, *p* < 0.0001), indicating progressive recovery (Table 8).

All candidate predictors had a VIF < 2.0, which indicated that multicollinearity was not significant (NIHSS = 1.02, age = 1.33, sex = 1.07, diabetes = 1.23, cardiomyopathy = 1.22, peripheral artery disease = 1.43, hyperlipidemia = 1.10, hypertension = 1.13, atrial fibrillation = 1.30, and depression = 1.02).

Through multivariable logistic regression, we showed that cardiomyopathy (CMP) and peripheral arterial disease (PAD) were significant predictors of upper limb recovery; presence of CMP was associated with lower odds of improvement (OR = 0.56; 95% CI: 0.28–0.96), while absence of PAD was associated with higher odds of improvement (OR = 2.15; 95% CI: 1.06–4.99).

## 4. Discussion

In this study, we investigated the impact of individual comorbidities on functional recovery following inpatient rehabilitation in patients after stroke. Consistent with previous research, significant improvements were observed across all functional domains, including activities of daily living (ADL), gait speed, balance, and upper limb function. However, the magnitude of recovery varied depending on the presence of specific comorbidities, highlighting their modifying role in rehabilitation outcomes.

Diabetes mellitus and depression emerged as significant negative determinants of ADL improvement, as measured by the Barthel Index (BI). Patients without diabetes had approximately 73% higher odds of functional improvement, while the absence of depression was associated with a more than twofold increase in improvement likelihood. These results align with prior studies demonstrating that metabolic dysfunction and affective disorders adversely influence post-stroke recovery [10,12,13]. Female sex was associated with lower odds of improvement in ADL, with an estimated reduction of approximately 30%. Chronic hyperglycemia may have impaired endothelial function, promoted inflammatory responses, and reduced synaptic plasticity, limiting rehabilitation-induced neuroplastic changes. Similar findings were reported by Karatepe et al. and Fischer et al., showing lower functional gains in diabetic stroke patients [10,24], and large observational studies have also indicated that diabetes is associated with slower recovery and reduced long-term independence [25].

Depression was one of the strongest predictors of poorer rehabilitation outcomes. In our study, depression was identified based on previously established clinical diagnoses and medical documentation, as well as on previously used therapies (SSRIs, antidepressants, etc.). Patients without depression had approximately 93% higher odds of improvement in gait speed and more than twice the odds of improvement in ADL. This is consistent with extensive research demonstrating that post-stroke depression negatively affects motor recovery, balance, and gait through behavioral and neurobiological mechanisms [26]. Reduced motivation, impaired concentration, and lower rehabilitation participation may partly explain these associations. The high prevalence of depression in our cohort is comparable to rates reported in European and global stroke populations, underscoring the need for systematic screening and multidisciplinary management in rehabilitation settings [27,28,29].

Gait speed improvement was influenced by stroke severity, age, sex, cardiomyopathy, and depression. Higher NIHSS scores were associated with 13% lower odds of gait speed improvement, emphasizing the critical role of initial neurological severity [30,31]. Older age was independently associated with reduced gait recovery, likely reflecting age-related declines in motor learning, muscle strength, and cardiovascular reserve [5,32]. Female patients exhibited approximately 51% lower odds of gait improvement compared with males and also demonstrated lower odds of improvement in ADL, with an estimated reduction of approximately 30%. These findings are consistent with previous meta-analyses reporting slower recovery trajectories in women, potentially due to older age at stroke onset, higher comorbidity burden, increased prevalence of depression, and social factors affecting rehabilitation access [33,34]. Cardiomyopathy was another key determinant, as patients without had more than twice the odds of gait speed improvement compared to those with cardiomyopathy. Reduced cardiac output and exercise tolerance in these patients may have limited engagement in intensive gait training [10,16].

Assessed by the Berg Balance Scale (BBS), balance recovery was significantly influenced by cardiomyopathy, as patients without had nearly twice the odds of improvement. Adequate cardiovascular function is essential for maintaining postural control, and cardiac limitations may restrict progression to more challenging exercises. Rizvi et al. demonstrated that adding cardiac rehabilitation exercises improved BBS outcomes compared with standard protocols, highlighting the negative impact of cardiovascular disease on postural stability and fall risk [35,36].

Measured by the Action Research Arm Test (ARAT), upper limb function was significantly affected by cardiomyopathy and peripheral arterial disease (PAD), with the former associated with lower odds of upper limb improvement, likely due to reduced endurance and training intensity [37]. Absence of PAD was associated with more than twice the odds of improvement, as vascular limitations may have impaired muscle perfusion and limited repetitive training efficacy [38,39].

Although hypertension and hyperlipidemia were prevalent in this cohort, neither emerged as independent predictors of functional recovery. While the former contributes to stroke pathogenesis, its direct influence on rehabilitation outcomes appears less significant when other factors, such as stroke severity, metabolic dysfunction, depression, and cardiac pathology, are considered [15,40]. Findings on hyperlipidemia are mixed, potentially reflecting differences in the study methodology, timing of assessments, and influence of statin therapy [41,42].

Specifically, the observed mean improvement in the established minimal clinically important difference (MCID) thresholds from baseline to discharge for the BI was 21.8 points (MCID: 10 points for BI), suggesting that the gains observed were not only statistically significant but represented a tangible improvement in the patients’ functional independence and safety [43]. The observed mean improvement in the BBS from baseline to discharge was +13.5 points, which exceeded the established MCID of 4 to 6 points for post-stroke populations [44], while for gait speed, the improvement was +0.32 m/s, which exceeded the established MCID of 0.10–0.20 m/s. This indicates that the functional gains observed were not only statistically significant but also clinically meaningful and likely to translate into real-world improvements in mobility and independence [45,46].

Overall, these results underscore the importance of comprehensive comorbidity assessment in stroke rehabilitation. While inpatient programs promote meaningful functional gains, the presence of specific comorbidities substantially modifies recovery potential. Optimal management of modifiable conditions such as diabetes and depression may enhance rehabilitation effectiveness, improve functional outcomes, and reduce long-term disability. Recognition of cardiovascular limitations should inform individualized rehabilitation planning, including exercise intensity adjustment and interdisciplinary collaboration.

This study has several limitations. First, its retrospective single-center design may have limited the generalizability of the findings and introduced potential selection bias; although the sample size was relatively adequate for an observational study, recruitment from a single rehabilitation institution where patients followed a standardized multidisciplinary program may have restricted the external validity of the results and their applicability to other healthcare systems or rehabilitation settings. The retrospective nature of the study restricted the availability of certain clinical and rehabilitation-related variables. Future prospective, multicenter studies incorporating more detailed clinical and rehabilitation-related variables are needed to further clarify the relationship between patient characteristics and outcomes after stroke. Second, comorbidities were assessed as binary variables (presence/absence) without information on disease severity, duration, or level of control, which may have resulted in residual confounding. Due to the retrospective nature of this study, these factors were not available in the primary dataset. In our next study, we will add a detailed section regarding these unmeasured confounders and how they might influence the observed associations between comorbidities and outcomes. Third, other factors that could have influenced rehabilitation outcomes—such as differences in therapy intensity and progression, therapist experience, patient adherence, social support, and baseline physical fitness—were not analyzed. Fourth, functional outcomes were only assessed during inpatient rehabilitation without long-term follow-up, limiting insight into sustained recovery. Given the observational design, the identified associations between comorbidities and functional outcomes should be interpreted as associative rather than causal. Finally, our analysis involved multiple pairwise comparisons, which may have increased the risk of type I error despite the application of statistical corrections. Therefore, the interpretation of interactions between comorbidity variables and time on functional outcomes should be approached with caution. Despite these limitations, in this study we provide valuable insights into the associations between common comorbidities and functional recovery during inpatient stroke rehabilitation, highlighting factors that may help guide individualized rehabilitation planning and optimize patient outcomes.

## 5. Conclusions

Inpatient rehabilitation significantly improved functional independence, gait speed, balance, and upper limb function in patients after stroke; however, recovery was strongly influenced by individual comorbidities and baseline clinical characteristics. Metabolic and psychological conditions, particularly diabetes mellitus and depression, were consistently associated with reduced gains in ADL and gait performance, while cardiovascular conditions, including cardiomyopathy and peripheral arterial disease, negatively affected gait, balance, and upper limb recovery. Additionally, higher stroke severity, older age, and female sex were associated with lower odds of gait improvement. These findings highlight that functional recovery after stroke is not solely determined by neurological impairment but is substantially modified by coexisting medical conditions. Integrated medical, psychological, and rehabilitative approaches are essential to optimize recovery and promote functional independence in stroke survivors.

## Figures and Tables

**Table 1:** Descriptive statistics of patients’ demographic characteristics, stroke characteristics, and comorbidities.

Variable	Categories	n (%)
Age (years)	<55	52 (18)
	55–65	88 (30)
	65–75	98 (34)
	>75	51 (18)
Sex	Male	167 (58)
	Female	122 (42)
Stroke lateralization	Right	157 (54)
	Left	121 (42)
	Both	11 (4)
Stroke localization	Cortical	69 (24)
	Subcortical	131 (45)
	Mixed	52 (18)
	Lacunar	13 (5)
	Cerebellum	24 (8)
Stroke type	Ischemic	254 (88)
	Hemorrhagic	35 (12)
NIHSS category	No symptoms	2 (1.0)
	Minor	88 (30.2)
	Moderate	193 (66.8)
	Severe	6 (2.0)
Diabetes mellitus (DM)	Yes	237 (82)
	No	52 (18)
Cardiomyopathy (CMP)	Yes	56 (19.4)
	No	233 (80.6)
Peripheral artery disease (PAD)	Yes	58 (20)
	No	231 (80)
Hyperlipidemia (HLD)	Yes	206 (71.3)
	No	83 (28.7)
Hypertension (HTN)	Yes	284 (98)
	No	5 (2)
Atrial fibrillation (AF)	Yes	46 (16)
	No	243 (84)
Depression	Yes	186 (64.5)
	No	103 (35.5)

Abbreviations: NIHSS, National Institutes of Health Stroke Scale; HTN, hypertension; CMP, cardiomyopathy; DM, diabetes mellitus; AF, atrial fibrillation; HLD, hyperlipidemia; PAD, peripheral arterial disease.

**Table 2:** One-way repeated-measures ANOVA with post hoc multiple-comparison tests for BI, gait speed, BBS, and ARAT.

Outcomes	Time Point	η_1_ ± sd_1_	η_2_ ± sd_2_	Test Statistics *	*p* Value *	F Value #	*p* Value #
	t1–t2	58.4 ± 31.4	70.1 ± 27	−15.6	<0.0001		
BI	t1–t3	58.4 ± 31.4	80.2 ± 24	−18.2	<0.0001	(367.6; 1.3) = 290.5	<0.0001
	t2–t3	70.1 ± 27	80.2 ± 24	−14.9	<0.0001		
	t1–t2	0.39 ± 0.36	0.55 ± 0.38	−13.6	<0.0001		
Gait speed	t1–t3	0.39 ± 0.36	0.71 ± 0.44	−14.2	<0.0001	(333.5; 1.3) = 245.7	<0.0001
	t2–t3	0.55 ± 0.38	0.71 ± 0.44	−16.9	<0.0001		
	t1–t2	29.3 ± 17.4	37.1 ± 15.6	−15.5	<0.0001		
BBS	t1–t3	29.3 ± 17.4	42.8 ± 14.7	−18.2	<0.0001	(339.5; 1.3) = 300.3	<0.0001
	t2–t3	37.1 ± 15.6	42.8 ± 14.7	−15.9	<0.0001		
	t1–t2	36.5 ± 23.7	40.6 ± 22.2	−7.65	<0.0001		
ARAT	t1–t3	36.5 ± 23.7	43.1 ± 21.7	−8.7	<0.0001	(323.5; 1.3) = 323.5	<0.0001
	t2–t3	40.6 ± 22.2	43.1 ± 21.7	−6.95	<0.0001		

**Abbreviations:** η ± SD, mean ± standard deviation; BI, Barthel Index; BBS, Berg Balance Scale; ARAT, Action Research Arm Test; t1 = baseline (upon admission), t2 = after three weeks of therapy, t3 = at discharge, η_1_ ± sd_1_—mean and standard deviation of 1, η_2_ ± sd_2_—mean and standard deviation of 2, *—test statistics and *p* value of multicomparison tests, #—test statistics and *p* value of the one-way repeated ANOVA.

**Table 3:** Distribution of Barthel Index categories across time points.

BI	t1 n (%)	t2 n (%)	t3 n (%)	*p* *	χ^2^ **
0–20	54 (19)	26 (9)	10 (4)	0.0004	15.8
21–40	89 (31)	70 (24)	54 (19)		
41–60	99 (34)	116 (40)	93 (32)		
61–80	20 (7)	35 (12)	45 (16)		
81–100	27 (9)	42 (15)	87 (30)		

* *p* value of Cochran’s Q tests; ** test statistics of Cochran’s Q tests.

**Table 4:** Multivariable logistic regression analysis for the Barthel Index.

Variable	β Coefficient	OR (95% CI)	*p* Value
Sex	−0.36	0.70 (0.43–0.94)	0.046
Diabetes mellitus	0.55	1.73 (1.09–3.46)	0.047
Depression	0.89	2.43 (1.98–3.68)	0.0005

**Table 5:** Distribution of gait speed categories across time points.

Gait Speed (m/s)	t1 n (%)	t2 n (%)	t3 n (%)	*p* *	χ^2^
>0.8	30 (12)	56 (22)	96 (38)	<0.0001	175
0.4–0.8	92 (36)	105 (41)	93 (37)		
<0.4	132 (52)	93 (37)	65 (25)		

* *p* value of Cochran’s Q tests; χ^2^, test statistics of Cochran’s Q tests.

**Table 6:** Multivariable logistic regression analysis for gait speed.

Variable	β Coefficient	OR (95% CI)	*p* Value
NIHSS	−0.13	0.87 (0.81–0.95)	0.002
Age	−0.12	0.88 (0.80–0.96)	0.046
Sex	−0.71	0.49 (0.28–0.85)	0.011
CMP	0.78	2.18 (1.05–4.76)	0.044
Depression	0.66	1.93 (1.78–2.38)	0.026

Abbreviations: NIHSS, National Institutes of Health Stroke Scale; CMP, cardiomyopathy; OR, odds ratio; CI, confidence interval.

**Table 7:** Berg Balance Scale categories across time points.

BBS	t1 n (%)	t2 n (%)	t3 n (%)	*p* *	χ^2^
0–20	84 (31)	50 (18)	32 (12)	<0.0001	203
21–40	95 (35)	79 (29)	43 (16)		
≥41	92 (34)	142 (53)	196 (72)		

* *p* value of Cochran’s Q tests; χ^2^, test statistics of Cochran’s Q tests.

**Table 8:** ARAT categories across time points.

ARAT	t1 n (%)	t2 n (%)	t3 n (%)	*p* *	χ^2^
0–20	68 (26)	52 (20)	45 (17)	<0.0001	98.3
21–40	91 (34)	91 (34)	68 (26)		
41–56	105 (40)	121 (46)	151 (57)		

* *p* value of Cochran’s Q tests; χ^2^, test statistics of Cochran’s Q tests.

## Data Availability

The original contributions presented in this study are included in the article. Further inquiries can be directed to the corresponding author.

## Abbreviations

The following abbreviations are used in this manuscript:

BI	Barthel Index
BBS	Berg Balance Scale
ARAT	Action Research Arm Test
NIHSS	National Institutes of Health Stroke Scale
CT	Computed Tomography
MRI	Magnetic Resonance Imaging
ADL	Activities of Daily Living
HTN	Arterial Hypertension
CMP	Cardiomyopathy
DM	Diabetes Mellitus
AF	Atrial Fibrillation
HLD	Hyperlipidemia
PAD	Peripheral Arterial Disease
AIC	Akaike Informtion Criterion
VIF	Variance Inflation Factor
EPV	Events Per Variable
MCID	Minimal Clinically Important Difference
